# Case report: Exploring cortico-muscular coherence during Mirror visual feedback for deafferentation pain: a proof-of-concept study

**DOI:** 10.3389/fnhum.2025.1525680

**Published:** 2025-02-24

**Authors:** Shiori Segawa, Michihiro Osumi

**Affiliations:** ^1^Graduate School of Health Science, Kio University, Nara, Japan; ^2^Neurorehabilitation Research Center, Kio University, Nara, Japan; ^3^Department of Rehabilitation, Hoshigaoka Medical Center, Osaka, Japan

**Keywords:** deafferentation pain, Mirror visual feedback, EEG, EMG, cortico-muscular coherence

## Abstract

**Background:**

Mirror visual feedback (MVF) has shown promise as a treatment for deafferentation pain following brachial plexus injury, yet the underlying mechanisms remain unclear. This study aimed to assess MVF’s effect on two patients with deafferentation pain by analyzing cortico-muscular coherence (CMC), a measure of functional connectivity between the brain and muscles.

**Methods:**

Two patients with brachial plexus injuries performed wrist movements with and without a mirror, accompanied by electromyography (EMG) and electroencephalography (EEG). CMC was calculated during each condition to determine changes in the sensorimotor network.

**Results:**

In Patient 1, CMC increased in the beta band in the extensor carpi radialis and surrounding parietal regions during the mirror condition. In Patient 2, beta-band CMC decreased in the compensatory muscle (biceps brachii) but increased in the primary muscle (flexor carpi ulnaris) when the mirror was used. These findings suggest MVF promotes sensorimotor integration, reducing pain intensity.

**Conclusion:**

Mirror visual feedback (MVF) effectively enhances CMC in the contralateral sensorimotor cortex in the beta frequency band, accompanied by pain relief in the affected limb. This suggests that CMC analysis could refine deafferentation pain rehabilitation using MVF, providing a better understanding of its neural mechanisms and optimizing therapeutic outcomes. Our study underscores the potential of CMC as a valuable biomarker for monitoring and tailoring MVF interventions.

## Introduction

The brachial plexus is a structure formed by five nerve roots (C5–T1) and is responsible for motor, sensory, and autonomic functions of the upper limb. Brachial plexus injury (BPI) can lead to pain associated with motor paralysis and sensory disturbances. This type of pain is referred to as deafferentation pain. Clinically, deafferentation pain is defined as a pathological pain that arises from lesions in the somatosensory pathways, leading to partial or complete loss of sensory input from specific body regions. It is commonly observed in conditions such as BPI and limb amputation (Hanakawa, 2012). Furthermore, the complex nature of deafferentation pain poses significant challenges in its treatment (Flor, 2002). Such motor paralysis and deafferentation pain can significantly reduce the quality of life (Shankar et al., 2015), requiring therapeutic strategies to alleviate symptoms (de Santana Chagas et al., 2022; Li et al., 2023). Mirror visual feedback (MVF), which uses visual illusions, has been proposed as a treatment for deafferentation pain (Mibu et al., 2016; Sumitani et al., 2008). In practice, a mirror is placed on the mid-sagittal plane of the subject’s body, with the hand of the non-paralyzed limb reflected in the mirror. By moving the non-paralyzed limb and either looking into the mirror while imagining that both limbs are moving symmetrically or moving the paralyzed limb to mirror the visual feedback of the non-paralyzed limb, the illusion is created that the paralyzed limb is moving like the non-paralyzed limb. It has been reported that this can alleviate deafferentation pain (Mibu et al., 2016; Sumitani et al., 2008). There are two types of MVF: one performed using motor imagery (Eren et al., 2024) and the other involving actual movements (Giraux and Sirigu, 2003). It has been reported that in MVF using extended reality (XR), both approaches are effective in reducing pain (Lendaro et al., 2024).

To date, numerous studies have been conducted to elucidate the mechanisms underlying MVF (Collins et al., 2018; Giraux and Sirigu, 2003; Hagenberg and Carpenter, 2014; Mercier and Sirigu, 2009). In patients with deafferentation pain, alterations in the sensorimotor loop have been reported (Shankar et al., 2015). It has been demonstrated that using motor illusions induced by visual feedback from MVF improves the sensorimotor loop following brachial plexus injury and alleviates deafferentation pain (Giraux and Sirigu, 2003; Mercier and Sirigu, 2009). Furthermore, reorganization of the primary motor cortex (M1) and primary somatosensory cortex (S1) has been identified as a key indicator of MVF’s efficacy (Foell et al., 2014; Giraux and Sirigu, 2003). In addition to M1 and S1, brain regions involved in multisensory integration, such as the fronto-parietal area, have also been reported to be recruited during MVF (Rizzolatti et al., 2009). Based on these findings, it is hypothesized that MVF facilitates the reorganization of M1 and S1 through afferent visual information transmitted via the fronto-parietal area (Rizzolatti et al., 2009). This process enhances the alignment between motor intent and sensory feedback, contributing to improved kinesthesia and pain relief.

However, the neural mechanisms underlying the therapeutic effect of MVF on deafferentation pain and the neuromuscular interactions during MVF rehabilitation remain unclear. To address this, we aimed to analyze the EEG signals and corresponding EMG signals simultaneously during MVF in patients with deafferentation pain after brachial plexus injury, assessing their synchrony - in other words, CMC. To date, numerous studies on cortico-muscular coherence (CMC) have been reported, along with review articles summarizing these findings (Brambilla et al., 2021; Gao et al., 2024; Lattari et al., 2010; Liu et al., 2019). CMC studies allow for the evaluation of the functional connectivity between the cerebral cortex and muscles, and it has been demonstrated that the generation of CMC reflects both descending neural information from the motor cortex to the muscles and ascending neural information from the muscles to the cerebral cortex (Lim et al., 2014; Witham et al., 2011). Also, since CMC involves frequency analysis, the interpretation of results varies depending on the frequency band in which brain and muscle activities are synchronized. Previous research has noted that beta-band coherence (13–30 Hz) increases in the contralateral sensorimotor areas during voluntary movement in healthy adults (Fang et al., 2009). This beta-band CMC is thought to reflect corticospinal tract activation associated with voluntary movement in humans (Fang et al., 2009). For example, reduced beta-band CMC has been reported in individuals who experience difficulty with coordinated movement, such as older adults, stroke patients, and those with amyotrophic lateral sclerosis (Krauth et al., 2019; Nielsen et al., 2008; Proudfoot et al., 2018). Given this background, our study aims to quantify changes in beta-band CMC with multi-channel EEG during MVF to elucidate the neural mechanisms associated with the analgesic effects of MVF on deafferentation pain. The mechanism of deafferentation pain is thought to involve a mismatch between the sensory feedback predicted by motor intention and the sensory feedback corresponding to the executed movement of the limb. This incongruence is believed to exacerbate pathological pain. In other words, the improvement of motor function is thought to generate appropriate sensory feedback in response to intentional movements, resolving the incongruence between motor intention and sensory feedback and thereby alleviating pain (Sumitani et al., 2008).

Based on the above findings, We hypothesize that the illusion induced by MVF improves motor function, alleviates deafferentation pain, and enhances beta-band CMC across widespread brain regions, including the contralateral sensorimotor area. Uncovering the brain-muscle interactions associated with MVF may provide crucial insights for developing new therapeutic approaches for patients suffering from deafferentation pain. Therefore, we conducted a preliminary analysis of CMC during MVF rehabilitation in two patients with deafferentation pain after brachial plexus injury.

## Methods

### Participants

We examined two patients with brachial plexus injury and severe deafferentation pain: Patient 1 (a 50 years-old man) had a lower brachial plexus injury, characterized by complete loss of somatosensation in the C8 dermatome (extending from the elbow to the distal ulnar side), with pain and a loss of somatosensory function in that area. Retaining shoulder and elbow mobility but limited voluntary wrist movement. He experienced severe pain spreading from the elbow to the hand and had previously undergone Dorsal Root Entry Zone (DREZ) lesioning surgery for pain relief. However, the surgery showed limited effectiveness. Additionally, he had received various physical therapies, such as Transcutaneous Electrical Nerve Stimulation (TENS) and neuromuscular facilitation, for pain management. Patient 2 (a 30 years-old woman) had an upper brachial plexus injury and had undergone the Oberlin procedure (Oberlin et al., 1994) to restore elbow flexion function. Regarding upper limb function, gradual improvement was observed following the Oberlin procedure. At the time of data measurement, although voluntary movements of the shoulder, elbow, and wrist were possible, it was limited. Furthermore, she received outpatient rehabilitation twice a week, including range-of-motion exercises, strength training, EMG biofeedback, and TENS.

Both patients had prior experience with MVF and had reported pain reduction after MVF. Before starting the experiment, we explained the study’s purpose and content to both participants and obtained written consent from each other.

In addition, two healthy adults participated in the study to provide control data.

### Procedure of Mirror visual feedback

**FIGURE 1:**
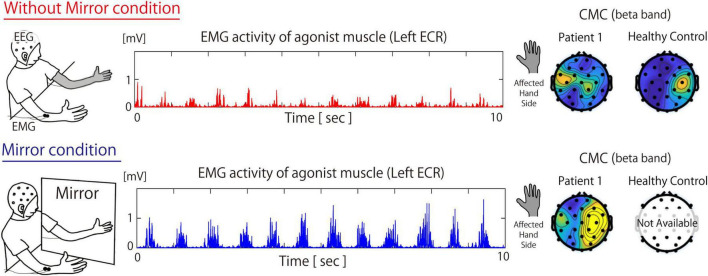
Measurement environment during the Without Mirror **(top)** and Mirror **(bottom)** conditions, muscle activity of the extensor carpi radialis, and the topographic map of the beta-band cortico-muscular coherence in patient 1 and Healthy participants. The topography for healthy participants is based on the grand average of data across all healthy participants. In the Mirror condition, both the activity of the primary agonist muscle (i.e., the ECR) and CMC increased. For the CMC data, under the Without Mirror condition, beta-band CMC in healthy participants was observed in the contralateral sensorimotor cortex relative to the target muscle. On the other hand, beta-band CMC of patient 2 in the contralateral sensorimotor cortex relative to the affected hand was not observed. However, under the Mirror condition, such beta-band CMC of Patient 1 was markedly increased in not only sensorimotor area but also the fronto-parietal area. ECR, extensor carpi radialis; CMC, cortico-muscular coherence; EEG, electroencephalography; EMG, electromyography.

Mirror visual feedbackinvolves the use of mirror reflections (Ramachandran and Rogers-Ramachandran, 1996). A mirror was placed along the mid-sagittal plane of participants’ bodies so that movements of the intact hand appeared as if they were being made by the affected hand (Figure 1 left). Each patient performed synchronous and periodic flexion-extension movements of both wrists in a quiet shielded room while seated comfortably in a stable chair. Initially, participants performed the movements without visual feedback (Without Mirror condition) while undergoing EEG and EMG. After a rest period, they were instructed to perform the same movements while receiving visual feedback via the mirror (Mirror condition). The task duration was 5 and 3 min for Patients 1 and 2, respectively. Given each patient’s residual motor function, Patient 1 was asked to perform wrist extension, whereas Patient 2 was asked to perform wrist flexion. The subjects were instructed to perform the movements at their own pace to avoid fatigue. Regarding the recording of two healthy participants, they performed wrist flexion-extension movements only under the Without Mirror condition because they had neither pain nor motor paralysis.

### EMG recording

Electromyography was acquired using a wired EMG system (Multipurpose Biological Amplification System MaP7810, Nihon Santeku Co., Ltd.) with customized electrode placements for each participant. To minimize skin resistance, the skin was prepped with alcohol wipes before electrode placement. For Patient 1, electrodes were placed on the extensor carpi radialis (ECR) bilaterally. For Patient 2, electrodes were placed on the flexor carpi ulnaris (FCU) and biceps brachii muscles (biceps). For healthy participants (*n* = 2), electrodes were placed on the extensor carpi radialis bilaterally. All EMG data were recorded at a sampling rate of 1,000 Hz during wrist extension and flexion movements. The recorded data were processed with an offline band-pass filter from 3 to 400 Hz.

### EEG recording

Electroencephalography was recorded using a 32-channel EEG amplifier (Active Two; BioSemi) at a sampling rate of 1,024 Hz. Signal^®^ Electrode Gel (Parker Laboratories, Fairfield, NJ, United States) was applied to each electrode to ensure proper signal transmission. Electrode placement followed the standard 10–20 system, with reference electrodes configured as CMS and DRL electrodes per BioSemi’s EEG setup. The recorded EEG signals were amplified and digitized across all channels. Offline data preprocessing involved resampling to 1,000 Hz using the EEGLAB toolbox in MATLAB R2021b (Mathworks) and applying a 1 Hz high-pass filter. Eye movement and EMG signal artifacts were removed using independent component analysis.

### Data processing and analysis

The CMC analysis was conducted using the extracted EMG and EEG data with MATLAB R2021b. First, muscle contraction peaks were detected from the EMG data of the target muscles. Then, an EMG data segment of 200 ms, centered around each peak (100 ms before and after), was extracted, along with the corresponding 32-channel EEG data. Each EMG and EEG data segment was concatenated to create a time series for analysis. CMC analysis was then performed using the extracted EMG and EEG data. The time window was set to 500 samples with an overlap of 100 samples. Coherence values were calculated using the following formula:


$$C\,M\,C_{S\,1,S\,2}\,\left(f\right)=\frac{{\left|P_{S\,1,S\,2}\,\left(f\right)\right|}^{2}}{\left|P_{S\,1}\,\left(f\right)\right|\times \left|P_{S\,2}\,\left(f\right)\right|}$$

where *P*_*S*1, *S*2_(*f*) represents the cross-spectral densities at a specific frequency (*f*), and *P*_*S*1_(*f*) and *P*_*S*2_(*f*) denote the power spectral densities of the EEG and EMG signals, respectively. The coherence function provides a normative scale of linear correlation on a scale of 0–1, where 1 indicates a perfect linear correlation, and 0 indicates no correlation.

The peak amplitude values of EMG were calculated as an indicator of motor function improvement in the cases, and a paired *t*-test was performed to assess significant differences between the Without Mirror and Mirror conditions. Significance was accepted at *p* < 0.05. Statistical analyses were performed using JASP software (version 0.19.1.0).

## Results

In both patients, deafferentation pain was immediately alleviated by MVF (from 8 to 4 in Patient 1 and from 7 to 5 in Patient 2 on a 10-point numerical rating scale). Both patients had the experience of seemingly being able to move their affected hands.

### Patient 1

Figure 1 shows the EMG and CMC data for Patient 1 in both the Without Mirror and Mirror conditions. In the Without Mirror condition, reduced muscle activity was observed in the ECR, while in the Mirror condition, ECR muscle activity increased (Figure 1, middle). The EMG results showed a significant difference (*p* < 0.05) in the peak amplitude of the ECR between the Without Mirror condition (0.226 ± 0.08 mV) and the Mirror condition (0.366 ± 0.094 mV). The CMC results showed the beta-band CMC was increased in not only the contralateral sensorimotor cortex but also surrounding regions when the mirror was introduced (Figure 1, right).

### Patient 2

**FIGURE 2:**
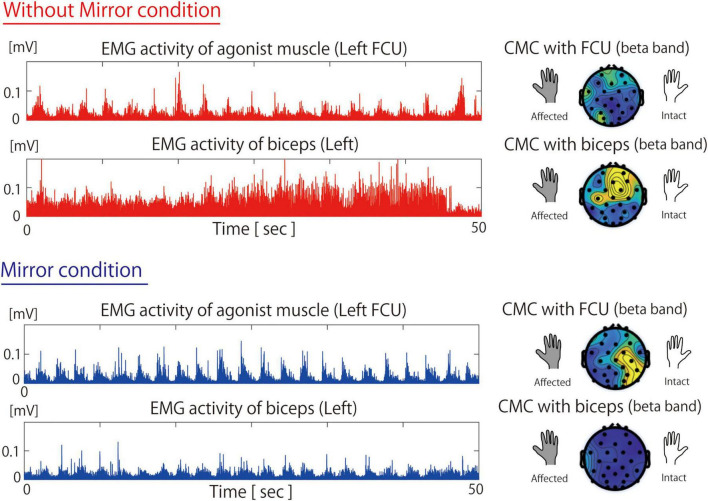
Muscle activity of the flexor carpi ulnaris and biceps and beta-band cortico-muscular coherence during the Without Mirror **(top)** and Mirror **(bottom)** conditions. In the Without Mirror condition, the non-primary agonist muscle (biceps) exhibited excessive activity and higher CMC. However, in the Mirror condition, these were reduced, which led to higher activity and CMC in the primary agonist muscle (FCU). FCU, flexor carpi ulnari; CMC, cortico-muscular coherence; EMG, electromyography.

Figure 2 shows the EMG and CMC data for Patient 2 in both the Without Mirror and Mirror conditions. In the Without Mirror condition, contraction-relaxation cycles of the FCU were observed, but the biceps brachii was overactive and remained in continuous contraction (Figure 2, middle). In contrast, in the Mirror condition, the overactivity of the biceps brachii was reduced, allowing the FCU to function as the primary active muscle for wrist flexion. The EMG results showed a significant difference (*p* < 0.05) in the peak amplitude of the biceps between the Without Mirror condition (mean ± SD: 0.032 ± 0.012 mV) and the Mirror condition (0.023 ± 0.009 mV). However, no significant difference was observed in the peak amplitude of the FCU between the Without Mirror condition (0.026 ± 0.009 mV) and the Mirror condition (0.041 ± 0.057 mV). The CMC results showed a higher beta-band CMC in not only the contralateral sensorimotor cortex but also the surrounding parietal regions in the Mirror condition than in the Without Mirror condition (Figure 2, right).

The EMG results showed a significant difference (*p* < 0.05) in the peak amplitude of the biceps between the Without Mirror condition (mean ± SD: 0.032 ± 0.012 mV) and the Mirror condition (0.023 ± 0.009 mV). However, no significant difference was observed in the peak amplitude of the FCU between the Without Mirror condition (0.026 ± 0.009 mV) and the Mirror condition (0.041 ± 0.057 mV).

### Healthy control

Under the Without Mirror condition, beta-band CMC between the ECR on the left side and EEG was calculated. As a result, a notable beta-band CMC coherence was observed in the contralateral sensorimotor region. This result reflects the typical topography of beta CMC in healthy individuals (Hallett et al., 2021).

## Discussion

In Patient 1, the activity of the agonist muscle and beta-band CMC was increased in not only the contralateral sensorimotor cortex but also surrounding regions when the mirror was introduced. Given that beta-band CMC indicates corticospinal tract activation, visual feedback during the Mirror condition might be associated with corticospinal tract activation and the contraction of the primary agonist muscle. The increase in beta-band CMC during MVF in both the contralateral sensorimotor cortex and surrounding regions has been reported in CMC studies involving stroke patients and is thought to occur during motor function recovery of the affected limb (Bao et al., 2018; Krauth et al., 2019). In healthy participants, beta-band CMC was localized to the contralateral sensorimotor cortex (i.e., right sensorimotor cortex) relative to the target muscle (i.e., left ECR). Unlike these data from healthy participants, CMC in Patient 1 under the Mirror condition showed an increase in beta-band CMC, centered in the sensorimotor area while also extending to the fronto-parietal regions. Considering that the fronto-parietal area is known as a brain region that integrates multisensory inputs and motor intentions (Desmurget and Sirigu, 2009; Desmurget and Sirigu, 2012; Desmurget et al., 2009; Perez et al., 2006; Vittersø et al., 2022), the increased beta-band CMC not only in the sensorimotor area but also in the fronto-parietal area under the Mirror condition suggests that visual information provided by the mirror might be associated with restoration of the disrupted sensorimotor integration and the alleviation of deafferentation pain.

Patient 2 underwent Oberlin surgery (Oberlin et al., 1994), whereby part of the ulnar nerve bundle was transferred to the musculocutaneous nerve to restore elbow flexion. Therefore, it is difficult to dissociate elbow flexion from wrist flexion. However, given that excessive muscle activity of the biceps brachii was reduced during MVF (Figure 2), the overactivity of the biceps brachii is not likely an irreversible change caused by the surgery. In this patient, appropriate visual feedback provided by MVF might be associated with the suppression of excessive biceps brachii activity and the activation of the primary agonist muscle responsible for wrist flexion. Because sensorimotor incongruence exacerbates deafferentation pain (Vittersø et al., 2022), deafferentation pain alleviation was likely achieved by optimizing the sensorimotor loop, as reflected by the CMC data. Reports have shown that beta-band CMC increases in the sensorimotor area reflect motor learning and visual feedback effects (Perez et al., 2006). Therefore, MVF-assisted feedback might optimize the sensorimotor loop between the primary agonist muscle and the contralateral sensorimotor cortex, which might be associated with motor learning and alleviation of deafferentation pain.

We measured beta-band CMC in two patients with deafferentation pain during MVF and observed improvements in the sensorimotor loop, beta-band CMC, and immediate deafferentation pain alleviation. While MVF technique has been reported as an effective rehabilitation approach for cases with deafferentation pain (Mibu et al., 2016; Sumitani et al., 2008), no studies have measured CMC during the MVF-based rehabilitation. Consequently, the evaluation of MVF’s effects has relied on subjective reports from individual patients. In the present study, we were the first to measure CMC during MVF-based rehabilitation in patients with deafferentation pain. We propose that its implementation in clinical settings could quantify the disturbed sensorimotor integration, and such quantification may contribute to advancing MVF-based rehabilitation strategies.

### Limitations

This study has several limitations in its experimental design.

First, data collection was limited to only two cases. To the best of our knowledge, no previous studies have measured CMC during MVF. Therefore, the observed changes in CMC are likely specific to these individuals, suggesting a strong influence of interindividual variability. Future studies should include a larger sample size to enhance the generalizability of findings.

Second, in the control group, data were collected only under the non-mirror condition, as no data were acquired for the mirror condition. The primary aim of this study was to measure CMC during muscle contraction, which requires the presence of actual muscle activity. In healthy individuals, motor performance is typically comparable between the mirror and non-mirror conditions, making the inclusion of both conditions less informative. To strengthen future experimental designs, additional conditions should be introduced. For example, conditions that deliberately manipulate visual feedback could be incorporated to simulate scenarios in which smooth motor performance is disrupted. Indeed, previous studies have evaluated visual feedback by introducing spatial (Don et al., 2019) or temporal (Imaizumi et al., 2014) distortions. Since this study lacked such technical manipulations, future research should incorporate specialized equipment to enable further validation.

Third, the two participants in this study had previously undergone MVF interventions and reported subjective benefits. Consequently, psychological factors may have influenced the outcomes, potentially introducing a placebo effect. To minimize such bias, future research should recruit participants with no prior exposure to MVF.

Fourth, peak amplitude changes in EMG were used as a surrogate marker for movement efficiency, without employing validated clinical scales or motion-tracking methods. This indirect measurement approach may not accurately reflect movement difficulty or functional capacity, potentially distorting the interpretation of MVF’s effects on motor function and pain reduction. Future studies should consider incorporating spatiotemporal indices from motion capture or inertial measurement unit (IMU) sensors, such as jerk analysis or trajectory assessments, to evaluate movement efficiency. Alternatively, if EMG is used, incorporating the co-contraction index may provide a more accurate assessment.

Fifth, in this study, all participants completed the mirror condition after the non-mirror condition, introducing a potential order effect. To mitigate this issue, future studies with larger sample sizes should employ counterbalancing or randomization in their experimental design.

Sixth, both participants in this study experienced pain. During the non-mirror condition, they may have focused attention on the affected limb, leading to pain anticipation and difficulty performing the task. In contrast, the mirror condition may have redirected their attention toward the reflection of the unaffected hand, alleviating fear and facilitating movement. This suggests that tasks designed to shift attention away from the affected limb, such as dual-task exercises, may also contribute to pain reduction. However, this study did not include specific control conditions to assess the role of attention, representing a limitation. Future studies should incorporate appropriate control conditions to account for the influence of attentional mechanisms.

Seventh, in cases of deafferentation pain following brachial plexus injury, patients often experience persistent pain, making it difficult to clearly differentiate between EEG components associated with abnormalities in sensorimotor integration and those specific to pain. To address this issue, future studies should include cases of somatosensory impairment without pain perception.

## Conclusion

The present study suggested that visual feedback might be associated with the restoration of the sensorimotor loop and the pain alleviation in patients with deafferentation pain. Notably, the key finding of the present study is that Mirror visual feedback (MVF) facilitates recovery not only by activating the sensorimotor cortex corresponding to the primary mover muscle but also through the integrative engagement of adjacent brain regions. This observation is consistent with the hypotheses proposed in previous studies (Foell et al., 2014; Giraux and Sirigu, 2003; Rizzolatti et al., 2009). However, to our knowledge, no previous studies have investigated the relationship between whole-brain neural activity and muscle activity in the context of MVF. Consequently, the present study provides a potentially novel contribution to elucidating the neural mechanisms underlying responsiveness to MVF. While this study is limited by the small sample size of two cases, these findings may hold clinical significance by informing the development of more targeted and effective rehabilitation strategies for this challenging condition.

## Data Availability

The raw data supporting the conclusions of this article will be made available by the authors, without undue reservation.
